# Severe Hyponatremia in the Setting of COVID-19-Associated Syndrome of Inappropriate Antidiuretic Hormone: A Case Report

**DOI:** 10.7759/cureus.33330

**Published:** 2023-01-03

**Authors:** Jorge A Gutierrez, David Ritzenthaler, Angeline Sawaya, Amanda L Pensiero

**Affiliations:** 1 Internal Medicine, Case Western Reserve University School of Medicine, Cleveland, USA; 2 Internal Medicine, University Hospitals Cleveland Medical Center, Cleveland, USA

**Keywords:** acute hyponatremia, covid 19, hyponatremia, syndrome of inappropriate antidiuretic hormone secretion, covid-19 retro

## Abstract

The COVID-19 pandemic has resulted in significant worldwide morbidity and mortality. One of the less studied clinical manifestations is Syndrome of Inappropriate Antidiuretic Hormone Secretion (SIADH) associated with COVID-19 pneumonia. We present a single case of COVID-19 pneumonia-associated SIADH in a 71-year-old male with a history of alcohol use disorder. This case highlights the importance of full diagnostic workup of the underlying cause of hyponatremia to avoid significant morbidity.

## Introduction

The COVID-19 pandemic has resulted in significant worldwide morbidity and mortality since the first cases were reported in December 2019. To date, over 600 million cases and over 6.5 million deaths have been confirmed [1]. This disease involves a myriad of organ systems which has sparked widespread research efforts in order to better understand and combat this aggressive pathology. One of the less studied clinical manifestations is hyponatremia in the setting of Syndrome of Inappropriate Antidiuretic Hormone Secretion (SIADH) associated with COVID-19 pneumonia.

SIADH is characterized by excessive release of antidiuretic hormone (ADH) independent of serum tonicity or blood volume. This results in water reabsorption in the kidneys and development of hypotonic hyponatremia [2]. Common etiologies of SIADH include central nervous system (CNS) disorders, malignancy, pulmonary disorders, and medications [3]. We present a single case of COVID-19 pneumonia-associated SIADH in a patient with chronic alcohol use which adds to the growing literature illustrating the impact of this disease on electrolyte balance.

## Case presentation

A 71-year-old male with a past medical history of hypertension, chronic obstructive pulmonary disease (not on home oxygen), depression, and alcohol use disorder with baseline consumption of roughly 1,025 mL of hard liquor daily initially presented to the emergency department at an outside hospital for altered mental status. In addition, he was hypoxic and chest X-ray noted bilateral primarily interstitial infiltrates consistent with COVID pneumonia. CT chest was negative for pulmonary embolism, but showed mid-lower lung predominant ground glass and consolidative pulmonary opacities. Bloodwork revealed a WBC count of 5.5, d-dimer 2180, and high-sensitivity C-reactive protein (CRP) 48.2. This data is more consistent with a viral rather than bacterial pneumonia. Hyponatremia (128 mmol/L) on admission was thought to be due to beer potomania and the patient was started on maintenance fluids. COVID infection was successfully treated over the course of three weeks with supplemental oxygen and complete courses of dexamethasone, remdesivir, and baricitinib.

The patient was subsequently transferred to our center for management of acute on chronic respiratory failure and persistent hyponatremia while awaiting placement at a skilled nursing facility. He was afebrile with a pulse of 64, blood pressure of 134/71, and respiratory rate of 18. Renal function panel on transfer showed hyponatremia (121/4.7/88/27/21/0.8/103). C-reactive protein was 48.2 and procalcitonin, ferritin, interleukin-6, lactate dehydrogenase, and d-dimer were not collected. Physical exam on arrival showed moist mucous membranes, no jugular venous distension, and intact distal capillary refill. The patient was initially given a 500-cc bolus of normal saline for suspected hypovolemia due to a recent illness which led to a decrease in serum sodium level to 115 mmol/L.

Additional labs were collected at this point showing a serum osmolality of 244 mmol/kg, urine osmolality of 690 mmol/kg, and urine sodium of 139 mmol/L. Thyroid-stimulating hormone (TSH), cortisol levels, and kidney function were all normal. No medical conditions or medications associated with SIADH were identified and a diagnosis of COVID-associated SIADH was made. The patient was treated with salt tablets and fluid restriction with close monitoring for overcorrection due to increased risk of central pontine myelinolysis. He was later switched to urea packets and furosemide for worsening hyponatremia with appropriate incrementation in sodium.

## Discussion

The COVID-19 pandemic has impacted everyday life throughout the globe. This disease has a highly variable presentation which makes correct diagnosis and early treatment a significant challenge. Hyponatremia in the setting of COVID-19 pneumonia is poorly studied and its pathogenesis incompletely understood at this point in time.

There are a number of factors that could potentially contribute to the inappropriate release of ADH in all pneumonias. Hypovolemia and hypotension frequently occur with pneumonia which can result in baroreceptor-mediated release of ADH [4]. The inflammatory cytokine IL-6 plays a major role in COVID-19 pneumonia and previous studies have cited it as likely contributing to the development of SIADH in this setting [5,6]. IL-6 stimulates nonosmotic release of ADH as well as damages the alveolar basement membrane, resulting in hypoxic pulmonary vasoconstriction and subsequent ADH release [7-10]. This ADH release leads to water reabsorption in the kidneys and the development of hypotonic hyponatremia [2]. This has been shown to be associated with significantly longer hospital stay [10] and increased risk of mortality [11,12].

The presented case highlights the importance of accurate diagnosis when managing electrolyte abnormalities. The drop in our patient’s sodium following an IV fluid bolus argued against beer potomania or dehydration as a potential cause. This led to subsequent lab workup with a significant elevation in urine osmoles and urine sodium. Additional labs and euvolemic exam findings ruled out hypothyroidism or a glucocorticoid deficiency pointing towards SIADH as the most likely etiology.

The first case of COVID-19-associated SIADH was reported in May 2020 [13]. While COVID-19 infection can present with a wide variety of symptoms such as fever, cough, myalgias, and dyspnea, clinical manifestations of hyponatremia can be the sole clinical presentation of COVID-19 infection and can potentially cloud diagnostic judgement [14]. Disorientation, confusion, seizures, stupor, and coma in the absence of respiratory symptoms could lead to misdiagnosis of COVID-19 and delay of treatment resulting in poor outcomes. While several case reports to date discuss this manifestation of COVID-19 pneumonia, none have described it in the setting of patients with underlying alcohol use disorder and cognitive impairment (Table 1) [13-21]. These underlying conditions leave the potential for anchoring and can result in a misdiagnosis of the patient’s illness.

**Table 1:** Comparison of COVID-associated SIADH Cases

Article	Age	Gender	COVID Pneumonia (present/absent)	Serum Sodium (nadir)	Serum Osmolarity	Urine Sodium	Urine Osmolarity	Underlying Alcohol Use Disorder	Underlying Cognitive Impairment/Dementia	Treatment	Clinical Outcome
Ho et al. [13]	75	M	present	104	230	58	693	No	No	hypertonic saline	survived
Habib, Sardar, Sajid [14]	57	M	absent	112	240	63	237	No	No	hypertonic saline	survived
Concepción Zavaleta et al. [15]	89	F	present	128	262	165	350	No	No	hypertonic saline, fluid restriction	survived
	70	M	present	124	254	175	372	No	No	hypertonic saline, fluid restriction	survived
Elmahal et al. [16]	54	M	absent	105	226	42	348	No	No	hypertonic saline	survived
Fernández Miró et al. [17]	52	M	present	127	268	77	1228	No	No	fluid restriction, hypertonic saline, hydroxychloroquine, azithromycin, ceftriaxone, lopinavir/ritonavir	survived
Habib et al. [18]	53	M	present	102	229	53	293	No	No	hypertonic saline, fluid restriction	survived
Kleybolte et al. [19]	86	M	absent	128	273	122	573	No	No	fluid restriction 1.2 L/day, d/c tramadol	survived
Sherazi et al. [20]	55	F	absent	116	251	69	364	No	No	hypertonic saline (first 24h), salt tablets, fluid restriction	survived
Tantisattamo et al. [21]	55	F	absent	120	255	28	326	No	No	Hydroxychloroquine, 0.9% NaCl	survived

## Conclusions

Our case of a patient who developed severe hypotonic hyponatremia with COVID-19 pneumonia is an important reminder of the importance of timely and appropriate diagnosis of the etiology of electrolyte imbalances in order to accurately manage these abnormalities. Physicians should remember to fully work up the cause of hyponatremia as treatment strategies differ based on etiology and significant morbidity can be avoided. Improper management can lead to catastrophic outcomes from severely depressed sodium.
